# Teaching hospital alternatives for Veterans Health Administration facilities: A Google Maps proximity study

**DOI:** 10.1371/journal.pone.0200219

**Published:** 2018-07-05

**Authors:** Joseph Bernstein, Loren Mead

**Affiliations:** 1 Surgical Services, Philadelphia Veterans Hospital, Philadelphia, PA, United States of America; 2 Perelman School of Medicine, University of Pennsylvania, Philadelphia, PA, United States of America; Aga Khan University, KENYA

## Abstract

The United States Veterans Health Administration (VHA) serves more than 9 million enrolled Veterans each year. Although most of the care that the VHA sponsors is delivered within its own facilities, there has been a call for “privatizing” some or all of these services. Under such an arrangement, the Department of Veterans Affairs would pay non-VHA providers to deliver care in facilities open to the general public. Privatization is hotly contested on political grounds and is not resolved. Yet the question whether the VHA should be privatized cannot be resolved without first establishing that this policy change is even feasible. One potential obstacle to privatization would be the lack of nearby alternative facilities to deliver care. To assess for the presence of this impediment, we used Google Maps to measure the travel time between 167 VA hospitals and the teaching hospital nearest to each of them. We determined that the mean travel time between VA hospitals and their nearest teaching hospital was approximately 18 minutes with a median of 10 minutes. All but nine VA facilities were within two hours’ travel, and these nine within ten minutes’ travel to a tertiary care, nonteaching hospital. These data do not definitively resolve the privatization debate, of course, but do refute the assertion that inpatient VA services cannot be privatized because replacement hospitals are too far away. As shown, that is simply not the case.

## Introduction

The healthcare system of the United States Veterans Health Administration (VHA) serves more than 9 million enrolled Veterans each year.

For some of the services it provides-mental health and primary care, for example-the quality of VHA facilities is said to exceed that of the private sector[1,2]. On the other hand, some patients seeking care within the VHA system have not received their services as expeditiously as they might have in non-VA facilities. Indeed, the Inspector General of the Veterans Administration (VA) testified to Congress that delays in care “had contributed to the deaths of patients” in at least one VA medical facility[3].

In response to the problem of delays, the Secretary of Veterans Affairs at the time, Dr. David Shulkin, was reported to favor expanding the department’s reliance on private health care for routine services. The benefit of this shift, Dr. Shulkin claimed, is that the VHA “can focus on its core mission of caring for the wounded.”[4] In a New York Times article on the subject, Dr. Shulkin responded to the report of a veteran who faced a long wait to get prosthetic replacements for legs lost in the line of duty by saying, “[w]e make eyeglasses for our veterans. Last time I checked, every shopping mall in America has a place where you can get glasses in an hour. I don’t care about making eyeglasses. I care about getting that veteran his prostheses.”[4]

But why stop at eyeglasses? There are many services provided by the VHA that lie outside of the core mission of caring for the wounded–services that are far more expensive than eyeglasses. The Veterans Health Administration hospitals provide inpatient care for cancer, coronary artery disease, arthritis and other medical conditions that are unlikely to be related to military service. Perhaps the VA might wish to purchase these forms of care as well.

The argument in favor of purchasing rather than providing specific forms of medical care was articulated by a former member of the United States Congress, John Linder, as follows: “There is a role for the government in veterans’ healthcare. [But it] is not the common cold, gall bladder surgery or dentures. Not even cancer. There are private facilities with newer tools and techniques that are better equipped for patient care…. The government’s role in veterans’ care should then be focused entirely on matters that are the result of war. Traumatic brain injury, amputations, post-traumatic stress and the rehabilitation from those injuries are unique and special, and we should dedicate the entire medical resources of our government toward improving the lives of the wounded and their families.”[5]

The position articulated by Linder has been termed the “privatization” of the VA: namely, a transition from providing care directly, by its own employees in its own facilities, to purchasing services offered on the open market to non-veterans. Of course, before one can determine whether nongovernmental hospitals *should* replace those of the Veterans’ Health Administration, one must first address the question of whether nongovernmental hospitals *could* replace them. In other words, there may be practical impediments to using nongovernmental hospitals as replacements that undermine any theoretical argument in their favor.

One practical impediment to using nongovernmental hospitals instead of VA hospitals is distance. That is, unlike what is seen in the case of eye-glasses (where alternative providers can provide their products in “every shopping mall in America”) replacement facilities to provide inpatient care for non-combat issues such as cancer, coronary artery disease, and arthritis may be simply too far away to serve as practical substitutes.

The research question we address, therefore, is how close are alternative sources for inpatient hospital services for veterans? Our null hypothesis is that for many veterans, there is no teaching hospital within one hour of the local VA hospital. This hypothesis, if not refuted, undermines the arguments in favor of “privatizing” the VA. On the other hand, if this hypothesis were to be refuted by showing that alternative teaching hospitals are nearby, one critical objection to the privatization efforts is similarly quashed.

## Methods

A list of VA “Health Care Systems” and “Medical Centers” was compiled using the 2018 *Directory of Federal Medical Treatment Facilities* as published by U.S. Medicine[6]. If a given Health Care System shared an address with a given Medical Center, only one entry was retained.

The number of veterans served at each of these facilities in FY 2017 was obtained from the Department of Veteran’s Affairs itself via a Freedom of Information Act (FOIA) request. The aggregation of these data allowed for the percentage contribution of each facility to the national workload to be calculated as well.

We obtained the locations of all US teaching hospitals as reported by the Centers for Medicare and Medicaid Services (CMS) [7].

Using Google Maps, we imported the locations of these facilities into a map via the “My Maps” feature. Several addresses reported in the official lists were unrecognizable when imported to My Maps (for example, PO boxes were not always recognized), and for them, a manual search by name was conducted.

Using a Google Map “pin” for each location represented on the map, we identified by eye the nearest teaching hospital for each VA facility. Driving directions were obtained between each VA facility and the nearby teaching hospitals by using the “Add directions” feature. We recorded the estimated driving time to the closest teaching hospital as reported by the “Show step-by-step directions” option of Google maps.

Using the travel times thus calculated, we were able to identify the set of VA hospitals that were farther than one hour from a teaching hospital. For these more-remote hospitals, we then conducted a Google search using the keywords “tertiary hospitals” and the name of the municipality and state in which the VA facility is located to identify the nearest tertiary care facility. Using the same Google maps procedure detailed above, the travel time between those remote VA hospitals and the nearest tertiary care facility was calculated.

## Results

There were 167 unique VA hospital facilities identified. According to the data provided by the VA, these facilities saw 7,559,770 total patients in FY 2017 (though some patients may have visited more than one facility and would be counted once at each).

The mean estimated travel time between VA hospitals and their nearest teaching hospital was 18.2 minutes; the median time was 10 minutes. (A complete list of the VA facilities and nearest teaching hospitals is given in S1 Appendix.)

**Fig 1:**
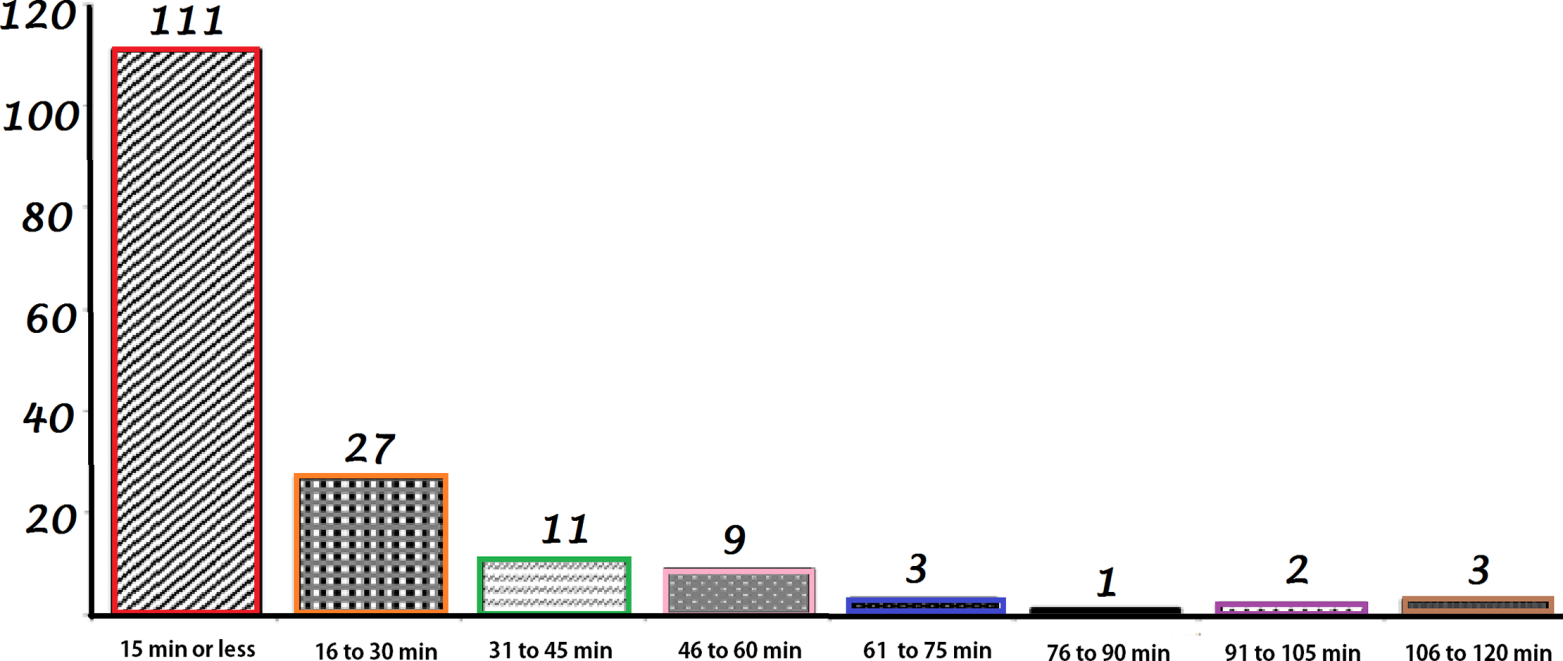
Distribution of VA hospitals as a function of the travel time to the nearest teaching hospital. As shown in this histogram, nearly all VA hospitals are within 60 minutes’ travel time.

The distribution of VA hospitals as a function of their travel time to replacement teaching hospitals is shown in Fig 1.

There were 9 teaching hospitals that were more than an hour from the nearest VA facility. These facilities saw 235,415 patients, approximately 3.1% of all patients seen. For these more-remote VA hospitals, a nearby replacement tertiary care center could be reached, on average, in 10 minutes.

In short, all VA hospitals were within 2 hours’ travel time of a replacement teaching hospital, and all VA hospitals were within 1 hours’ travel time of a replacement teaching hospital or tertiary care facility.

## Discussion

There is a broad consensus in America that veterans should receive healthcare services paid for by the US government. There is less agreement regarding where these services should be provided. Although at present the VA delivers almost all care within its own facilities, there are strong proponents for the argument that the VA should be “privatized”: namely, that the VA should purchase healthcare, but not provide it directly. Under this approach, veterans would receive a voucher or similar award that allows them to pay for healthcare, or healthcare insurance, in the public sector.

There are international precedents for privatizing the care given to military veterans. Even with the United States, the VA has historically offered some care outside the confines of its own facilities. The Hometown Medical Care program initiated in 1945, for example, paid for private medical services. In 1958, Congress passed Title 38 of the United States Code that allowed for the purchase of non-VA health services “when VA medical facilities cannot provide services economically due to geographical inaccessibility, or in emergencies when delays may be hazardous to a veteran’s life or health”[8]. More recently, the Veterans Choice Program allowed eligible patients to receive health care from a community provider if they were facing a wait greater than 30 days for an appointment or were living more than 40 miles from the closest VA medical facility with a full time primary care physician.

Of course, these programs attempt only to supplement, not supplant, the VA. Still, there are those who prefer to take the outside-the-system approach to its extreme. For them, the correct choice is to close the VA hospitals and provide funds for veterans to obtain their care in the conventional market[9].

Opponents of privatization cite the unique attributes of VA hospitals and the preference of veterans themselves. Yet perhaps the most compelling argument against closing VA hospitals is that, for many veterans, there simply is no nearby replacement hospital. If the VA hospitals were to close, this argument goes, the veterans will be left with nowhere to go.

The purpose of our study is to scrutinize that contention.

The specific aim of our study is to calculate the travel time between all VA hospitals and the nearest neighboring (non-VA) teaching hospital. We measured this travel time using Google maps. Our results reveal that for many veterans a replacement is indeed not far. As shown, the typical VA hospital is only approximately 10 minutes away from a teaching hospital replacement, and well over 95% of VA hospital visits were at facilities that are within one hour of a replacement.

These results demonstrate that it is not reasonable to claim that VA hospitals cannot be closed because suitable replacements are too far away. Alternatives are indeed nearby.

It is not surprising that many alternative facilities were close at hand. In fact, this proximity is by design. Following World War II, the VA faced a vast shortage of clinicians to meet the needs of 16 million demobilized troops. Accordingly, the Director of VA Medicine, Dr. Robert F. Hawley, signed Policy Memorandum #2, which provided for the creation of these associations with medical schools[10]. VA affiliations with medical schools flourished, with 70 VA hospitals located within 5 miles of a medical school by 1980. Given that many medical schools share their campus with an affiliated teaching hospital, the proximity we discovered should not be unexpected.

It is further likely that our results overstate the travel time needed to find a replacement hospital. Our set of alternatives was taken from a list of teaching hospitals, and the criteria for inclusion on that list omits some hospitals that may indeed be able to provide most, if not all, of the necessary services. Moreover, as noted, the nine hospitals that were more than one hour away from a teaching hospital were still quite close to a tertiary care [but not teaching] hospital.

The results presented here may also be useful to The Department of Veterans Affairs itself, when it considers the appropriate remedy for facilities that are performing poorly. To be sure, closure of such facilities may be more easily contemplated if replacement facilities are nearby.

Our results provide an upper-bound on the additional travel time needed, and indeed for some patients, the replacement facility might even be closer. Without access to information about where each individual patient resides, it is impossible to calculate the true average change in travel time, but it is likely that the additional travel time is much less than the maximum.

### Limitations

The major limitation of this study is that its methods address replacement facilities for hospital care only. Most of the service delivered by the VA is primary care, and that was simply not studied here. Thus, it would not be reasonable to claim, based on the findings here, that the entire VA can be privatized; rather, the assertions would have to be limited to privatizing in-patient services alone. In terms of the formulation of John Linder, cited above, the study here does not address the VA’s role for treating the common cold, but it might very well apply to gall bladder surgery.

The use of Google maps might be another weakness of the study. To be sure, our results can be no more valid than the information provided by Google. That said, there is no particular reason to find the data suspect. Also, to the extent that our “by eye” method may have missed a closer alternative teaching hospital, the error would tend to overstate the distance, and, if anything, strengthen the conclusion.

In addition, we would argue that any imprecision introduced by using Google is more than offset by how it liberalizes health services research. For example, a study that considers the home addresses of all VA patients and calculates mean per-person additional travel time will yield results that are more robust than can be had with our methods. Nonetheless, only the VA itself could perform such a study, and to our knowledge it has not. Armed only with Google (and FOIA), amateurs without funding, as we are, can offer analyses that in the past were likely limited to insiders. Thus, if insiders are reluctant to publish findings contrary to current policy, an imprecise Google study, flaws notwithstanding, advances knowledge overall.

Another limitation of our methods is that there are no assurances that the nearby teaching hospital necessarily can offer all of the services that are offered at the VA hospital itself. After all, there are teaching hospitals that do not offer every conceivable service. On the other hand, it is possible that the VA hospital itself is deficient in some area, and that the replacement hospital actually offers a broader range of services. This issue can be resolved only be a more granular analysis that what is given here. As a confidence measure, we found (using the same methodology as above) that VA hospitals were a median 21 minutes from the nearest medical school campus–the site of broadest care service in the neighborhood, one can assume. Thus, even if the identified replacement teaching hospital were deemed deficient, that gap can likely be filled with scant additional travel time.

It must be kept in mind that the mere presence of a facility is not sufficient proof that a reasonable alternative is at hand. A valid replacement facility must have the capacity to provide the necessary services; it must deliver these replacement services with high quality and at a fair price; and it must have systems of accountability to ensure that high-quality care will endure. Thus, the VA must have the opportunity to scrutinize outcomes and ensure value for what it pays. To that end, it may be necessary to have not only one replacement facility nearby, but perhaps two or more, sufficient to promote competition. It is unknown how many VA facilities will be within adequate distance of potential replacements that meet this requirement. This, too, demands further study.

### Conclusion

The question about privatizing the VA is hotly contested. There are reasonable arguments on both sides. We make no assertion that we have definitively resolved this debate. Indeed, we have not. Our sole claim is that we have refuted the assertion that inpatient VA services cannot be privatized because replacement hospitals are too far away. As shown, that is simply not the case.

## Supporting information

S1 Appendix (XLSX)
